# The connection between tricarboxylic acid cycle enzyme mutations and pseudohypoxic signaling in pheochromocytoma and paraganglioma

**DOI:** 10.3389/fendo.2023.1274239

**Published:** 2023-10-05

**Authors:** Yuxiong Wang, Bin Liu, Faping Li, Yanghe Zhang, Xin Gao, Yishu Wang, Honglan Zhou

**Affiliations:** ^1^ Department of Urology, The First Hospital of Jilin University, Changchun, Jilin, China; ^2^ Key Laboratory of Pathobiology, Ministry of Education, Jilin University, Changchun, Jilin, China

**Keywords:** pheochromocytoma, paraganglioma, pseudohypoxia, VHL/HIF axis, genetic mutations, tricarboxylic acid cycle, metabolic reprogramming

## Abstract

Pheochromocytomas and paragangliomas (PPGLs) are rare neuroendocrine tumors originating from chromaffin cells, holding significant clinical importance due to their capacity for excessive catecholamine secretion and associated cardiovascular complications. Roughly 80% of cases are associated with genetic mutations. Based on the functionality of these mutated genes, PPGLs can be categorized into distinct molecular clusters: the pseudohypoxia signaling cluster (Cluster-1), the kinase signaling cluster (Cluster-2), and the WNT signaling cluster (Cluster-3). A pivotal factor in the pathogenesis of PPGLs is hypoxia-inducible factor-2α (HIF2α), which becomes upregulated even under normoxic conditions, activating downstream transcriptional processes associated with pseudohypoxia. This adaptation provides tumor cells with a growth advantage and enhances their ability to thrive in adverse microenvironments. Moreover, pseudohypoxia disrupts immune cell communication, leading to the development of an immunosuppressive tumor microenvironment. Within Cluster-1a, metabolic perturbations are particularly pronounced. Mutations in enzymes associated with the tricarboxylic acid (TCA) cycle, such as succinate dehydrogenase (SDHx), fumarate hydratase (FH), isocitrate dehydrogenase (IDH), and malate dehydrogenase type 2 (MDH2), result in the accumulation of critical oncogenic metabolic intermediates. Notable among these intermediates are succinate, fumarate, and 2-hydroxyglutarate (2-HG), which promote activation of the HIFs signaling pathway through various mechanisms, thus inducing pseudohypoxia and facilitating tumorigenesis. SDHx mutations are prevalent in PPGLs, disrupting mitochondrial function and causing succinate accumulation, which competitively inhibits α-ketoglutarate-dependent dioxygenases. Consequently, this leads to global hypermethylation, epigenetic changes, and activation of HIFs. In FH-deficient cells, fumarate accumulation leads to protein succination, impacting cell function. FH mutations also trigger metabolic reprogramming towards glycolysis and lactate synthesis. IDH1/2 mutations generate D-2HG, inhibiting α-ketoglutarate-dependent dioxygenases and stabilizing HIFs. Similarly, MDH2 mutations are associated with HIF stability and pseudohypoxic response. Understanding the intricate relationship between metabolic enzyme mutations in the TCA cycle and pseudohypoxic signaling is crucial for unraveling the pathogenesis of PPGLs and developing targeted therapies. This knowledge enhances our comprehension of the pivotal role of cellular metabolism in PPGLs and holds implications for potential therapeutic advancements.

## Introduction

Pheochromocytomas (pcc) and paragangliomas (PGL) (PPGLs) are infrequent neuroendocrine tumors deriving from chromaffin cells. Pheochromocytoma originates in the adrenal medulla, accounting for approximately 80%-85% of cases, while paraganglioma arises externally from sympathetic paraganglia dispersed throughout the body, constituting around 15%-20% of cases (1–3). Despite their low incidence rate (roughly 6.6 cases per million individuals annually) (4), these tumors bear noteworthy clinical significance due to their potential to induce excessive catecholamine secretion, precipitating perilous cardiovascular complications and myocardial degenerative alterations. In the latest World Health Organization guidelines, all PPGLs are now recommended for lifelong follow-up due to their metastatic potential, similar to epithelial neuroendocrine tumors (5–7).

Approximately 80% of PPGLs are linked to genetic mutations, often indicating a genetic predisposition (2, 8). Gene expression profiling enables the classification of PPGLs into three distinct clusters based on their molecular characteristics: the pseudohypoxia signaling cluster (Cluster-1), the kinase signaling cluster (Cluster-2), and the WNT signaling cluster (Cluster-3). Cluster-1 further divides into subclusters 1a and 1b. Cluster-1a comprises mutations in genes associated with the tricarboxylic acid cycle (SDHx, FH, MDH2, GOT2, SLC25A11, IDH, DLST, SUCLG2), while Cluster-1b encompasses mutations in genes constituting the hypoxia signaling pathway (PHD1/2, VHL, HIF2A/EPAS1) (9–11). Cluster-1 notably exhibits a significant enhancement of pseudohypoxic signaling pathways, resulting in heightened angiogenesis and metabolic anomalies during tumor development. Cluster-1a represents approximately 10%-15% of cases, while Cluster-1b accounts for approximately 15%-20% of PPGLs. Importantly, Cluster-2 within the PPGLs group demonstrates a marked increase in gene expression related to kinases. This involves critical mutations in genes associated with the PI3K/mTORC1 pathway and receptor kinase signaling, such as RET, NF1, H-RAS, K-RAS, TMEM127, and MAX, implying their potential significance in regulating tumor growth and metastasis. Cluster-2 constitutes approximately 50%-60% of PPGLs cases. Cluster-3 is characterized by the activation of the WNT signaling pathway, potentially contributing to increased cellular proliferation and invasiveness. Notably, Cluster-3 accounts for approximately 5%-10% of PPGLs cases and also includes tumors with mutations in the CSDE1 and the MAML3 fusion genes (2, 12, 13). In the three clusters, Cluster 1 exhibits the most prominent risk of metastasis (9). Additionally, among the cases in Cluster 1, there is typically an elevation in norepinephrine levels, which is associated with a deficiency in phenylethanolamine N-methyltransferase (13). This results in a tendency for such patients to have persistent high blood pressure (14). These findings underscore our continued focus on Cluster 1.

Roughly 40% of PPGLs patients carry a germline mutation in one of over 20 known susceptibility genes. In an additional 30-40% of sporadic disease patients, somatic mutations in the same genes or other genes that drive tumorigenesis can be identified (14, 15). Multiple studies indicate that Cluster 1a is almost exclusively germline-mutated (100%), while Cluster-1b has 25% of cases with germline mutations (13, 16). Among PPGLs, the top three genes with germline mutations are SDHB (10.3%), SDHD (8.9%), and VHL (7-10%), while the genes with the highest somatic mutation frequencies are VHL (10%) and HIF2A (5-7%), with germline or somatic mutations in other genes being less than 2% (15, 17–19). This highlights the significant role of germline mutations in the pathogenesis of Cluster-1 PPGLs. Clinical studies on sporadic PPGLs show a significantly higher occurrence of germline mutations associated with multiple PPGLs compared to isolated PPGLs (54% *vs*. 11.5%). Moreover, the research suggests that the risk of germline mutations in PPGLs located outside the adrenal gland is significantly higher than those within the adrenal gland (14, 20). Specifically for Cluster 1, PPGLs associated with mutations in TCA metabolic enzyme genes (Cluster-1a) are mainly located outside the adrenal gland. For example, SDHA mutations are linked to sympathetic and parasympathetic PGLs (21). SDHB and SDHC mutations are primarily observed in sympathetic/parasympathetic PGLs, but are less common in PCC (22). SDHD and SDHAF2 mutation-related PGLs are predominantly found in the head and neck region, with lower incidence in other extrarenal locations or within the adrenal gland (23, 24). Tumors associated with FH mutations are found both inside and outside the adrenal gland. In Cluster-1b, VHL mutations causing PCC result in 50% of cases being bilateral and occasionally occurring outside the adrenal gland. HIF2A/EPAS1 mutations may lead to tumors in both intra- and extra-adrenal locations (14).

The hypoxia-inducible factor-2α (HIF2α) protein gets upregulated in cells under normal oxygen conditions, culminating in the activation of downstream transcriptional processes, recognized as pseudohypoxia. Activation of the hypoxia response pathway confers advantages to tumor cell growth and adaptation to adverse microenvironments (25). Studies have elucidated heightened pseudohypoxic signaling in hereditary renal cell carcinoma, giving rise to the upregulation of downstream signaling molecules such as glucose transporter 1 and vascular endothelial growth factor (VEGF), thereby fostering augmented energy metabolism and proliferative capacity in tumor cells (26). Pseudohypoxic signaling prompted by tumor cells modifies the communication patterns of immune cells, instigating alterations in immune cell metabolism that incline towards the attenuation of surveillance function in innate immune cells and the fostering of an immunosuppressive microenvironment (25, 27). Specimens of neuroblastoma and glioma have evinced the prevalence of elevated HIF2α expression and a concomitant display of nascent characteristics, signifying the presence of a pseudohypoxic niche in these tumors that correlates with malignancy (28). The instigation of pseudohypoxia through HIFs signaling also plays a pivotal role in non-neoplastic ailments. The buildup of reactive oxygen species (ROS) in lung tissue engenders HIFs activation in fibroblasts, disrupting the structural integrity of the extracellular matrix and exacerbating the progression of pulmonary fibrosis (29). In the course of the aging process, diminished nuclear NAD+ levels impede the activity of nuclear SIRT1, thus suppressing the pVHL ubiquitin-proteasome degradation pathway. This engenders heightened stability of HIF1α within the cell and the concomitant emergence of HIF1α-induced pseudohypoxia. The pseudohypoxic state engendered by HIF1α disrupts intracellular signaling between the nucleus and mitochondria, thereby impairing mitochondrial function and accelerating cellular senescence and demise (30).

Pseudohypoxia has been meticulously scrutinized and observed in the context of PPGLs. Notwithstanding their typical highly vascularized nature, PPGLs are characterized by a conspicuous upregulation of hypoxia signaling pathways, particularly within Cluster-1, thereby fueling the onward progression of tumors (25, 31–33). The principalstay treatment for PPGLs remains surgical intervention, as effective therapeutic modalities for metastatic tumors are presently scarce (34). A profound comprehension of the hypoxia-related signaling mechanisms underpinning PPGLs pathogenesis is imperative for the development and assessment of molecularly targeted therapies tailored to the diverse subtypes of PPGLs, encompassing the intricacies of the tumor microenvironment (TME). This comprehensive review seeks to illuminate how mutations in metabolic enzymes within Cluster 1a of the tricarboxylic acid cycle impinge upon the VHL/HIF signaling pathway, consequently contributing to the genesis of PPGLs. This dissection facilitates an enhanced understanding of the pivotal role of cellular metabolism in the realm of PPGLs, thereby engendering substantial implications for the refinement of extant therapeutic modalities.

## Dysregulation of the VHL/HIF signaling axis in PPGLs

**Figure 1:**
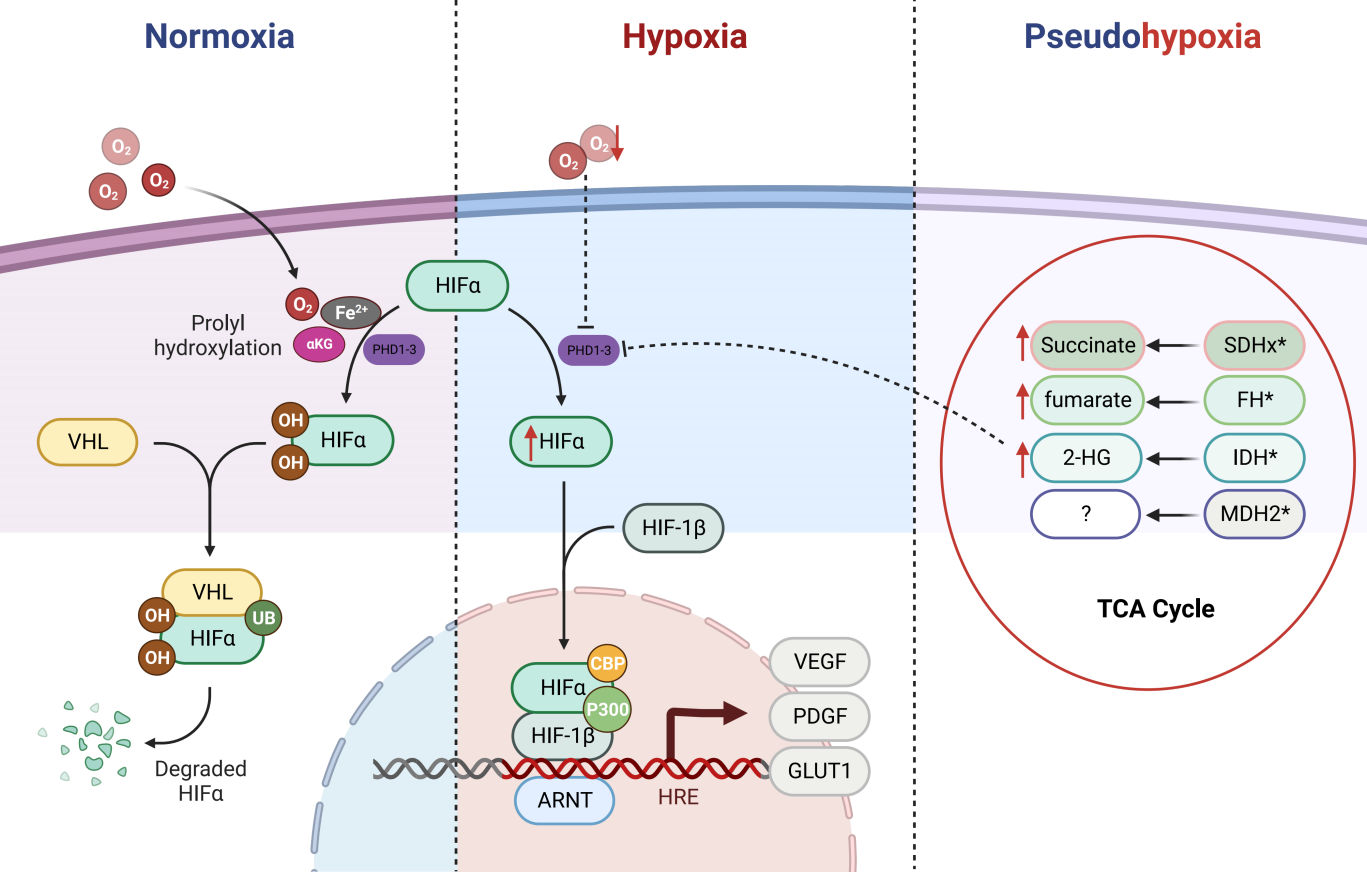
Regulation of HIFα (HIF-1α/HIF-2α) by the PHD/VHL Pathway and Impact of Tricarboxylic Acid Cycle Metabolic Mutations. HIFα (HIF-1α/HIF-2α) is primarily regulated by the PHD/VHL pathway. Under normoxic conditions, the prolyl hydroxylase domain (PHDs) proteins act upon the prolyl residues of HIFα in the presence of cofactor oxygen molecules, αKG, and Fe^2+^, leading to hydroxylation. The hydroxylated HIFα is subsequently recognized and bound by E3 ubiquitin ligase VHL, followed by ubiquitination and subsequent degradation as a substrate within the proteasome. Under hypoxic conditions, the function of PHDs is inhibited, resulting in direct accumulation of HIFα. Subsequently, HIFα forms a heterodimer with HIF-1β and translocates into the nucleus, where with the assistance of p300/CBP, it activates downstream oncogenic signaling pathways. In cases of mutations in the TCA cycle metabolic enzymes (SDHx, FH, IDH, and MDH2), metabolic intermediates such as succinate, fumarate, and 2-HG accumulate. Due to their structural similarity to αKG, they competitively inhibit the αKG-assisted activation of dioxygenases (such as PHDs). As a result, under normoxic conditions, HIFα accumulates and activates downstream pathways, leading to a pseudohypoxic state.

The intricate interplay between the VHL tumor suppressor and HIFs signaling axis bears a profound relevance to the emergence of PPGLs (35, 36). Central to this dynamic is the orchestration of HIFs’ stability by oxygen concentration within the microenvironment (see **Figure 1**). Under normoxic conditions, prolyl hydroxylation of HIF1α/HIF2α by oxygen-dependent prolyl hydroxylases (PHDs) triggers subsequent recognition by the E3 ubiquitin ligase VHL protein. This culminates in selective ubiquitination and proteasomal degradation of hydroxylated HIFα subunits. Conversely, under hypoxia, PHDs activity inhibition leads to non-hydroxylated HIFα subunit accumulation. Following nuclear translocation, heterodimerization with HIF1β ensues, facilitating transcriptional complex formation with coactivators p300/CBP (25, 37). This complex binds to hypoxia response elements, activating transcription of genes such as VEGF, platelet-derived growth factor (PDGF), and glucose transporter (GLUT) (11, 38, 39). Such adaptations promote metabolic reprogramming in hypoxic cells, characterized by elevated glucose uptake, anaerobic glycolysis, diminished mitochondrial mass, and heightened energy provisioning, thus fostering tumorigenic progression. Tumors frequently exhibit an intriguing phenomenon whereby normoxic conditions lead to an anomalous stabilization of HIFs within tumor cells. This multifaceted phenomenon results from diverse influences, ultimately culminating in the accumulation or heightened functional activity of HIFs. Consequently, this dynamic process upregulates the expression of HIFs target genes, remarkably mimicking the pathophysiological response seen in hypoxic states. Coined as “pseudo-hypoxia,” this intriguing state prompts notable cellular transitions, including epithelial-mesenchymal transition, augmented tumor cell stemness, thereby effectively fueling the cascade of tumorigenic initiation, progression, and the potentiation of malignant attributes (40, 41).

The regulatory apparatus governing the VHL/HIF axis is intricate, encompassing a myriad of factors. Within the landscape of sporadic PPGLs, a significant subset, approximately 14%, manifests somatic VHL mutations (42). While the precise mechanistic underpinnings characterizing VHL dysfunction and its structural aberrations in the pathogenesis of PPGLs remain enigmatic, a discernible association between missense mutations in the VHL gene, particularly at positions 167 and 238, and the occurrence of PPGLs is evident (43). The implications of such missense mutations or reduced VHL expression are profound, manifesting in the impediment of HIFα ubiquitination and degradation. Consequently, a stabilization of HIFs transpires, subsequently engendering an augmented susceptibility to the development of PPGLs, specifically implicating Type 2 VHL disease (44–46). The landscape of HIFs genetic alterations is further nuanced, with HIF2α mutations assuming a more pronounced presence. Notably, germline mutations in HIF2A exon 9 (c.1121T>A, p.F374Y) significantly enhance the propensity for PPGLs (47). Comparatively, PHD mutations within the realm of PPGL patients present as a relatively infrequent occurrence, a notable distinction in contrast to the prominent roles of VHL and HIFα (48).

Intriguingly, PPGLs are increasingly recognized as metabolic disorders, particularly within the spectrum of cluster 1a manifestations. The manifestation of mutations within tricarboxylic acid (TCA) cycle enzymes precipitates an accumulation of pivotal metabolic intermediates. Subsequently, a multipronged cascade is set in motion, activating the HIFs signaling pathway via diverse mechanisms, thus engendering a pseudo-hypoxic milieu. Elevated levels of metabolites such as succinate, fumarate, or 2-hydroxyglutarate (2-HG) are indicative hallmarks within PPGLs, closely mirroring mutations inherent to TCA cycle enzymes (49–51). These metabolites, intricately interwoven with the tumorigenic context, proceed to exert their influence by modulating HIFs activity or by influencing the regulatory cascade upstream, thereby orchestrating the transduction of oncogenic signals. Within this framework, our review assumes a focal orientation, dedicated to the elucidation of the nuanced impact instigated by mutations within four pivotal TCA cycle enzymes—namely, Succinate dehydrogenase (SDHx), fumarate hydratase (FH), isocitrate dehydrogenase (IDH), and malate-dehydrogenase type 2 (MDH2)—upon the intricate VHL/HIF axis in PPGLs.

## The TCA cycle and PPGLs

**Figure 2:**
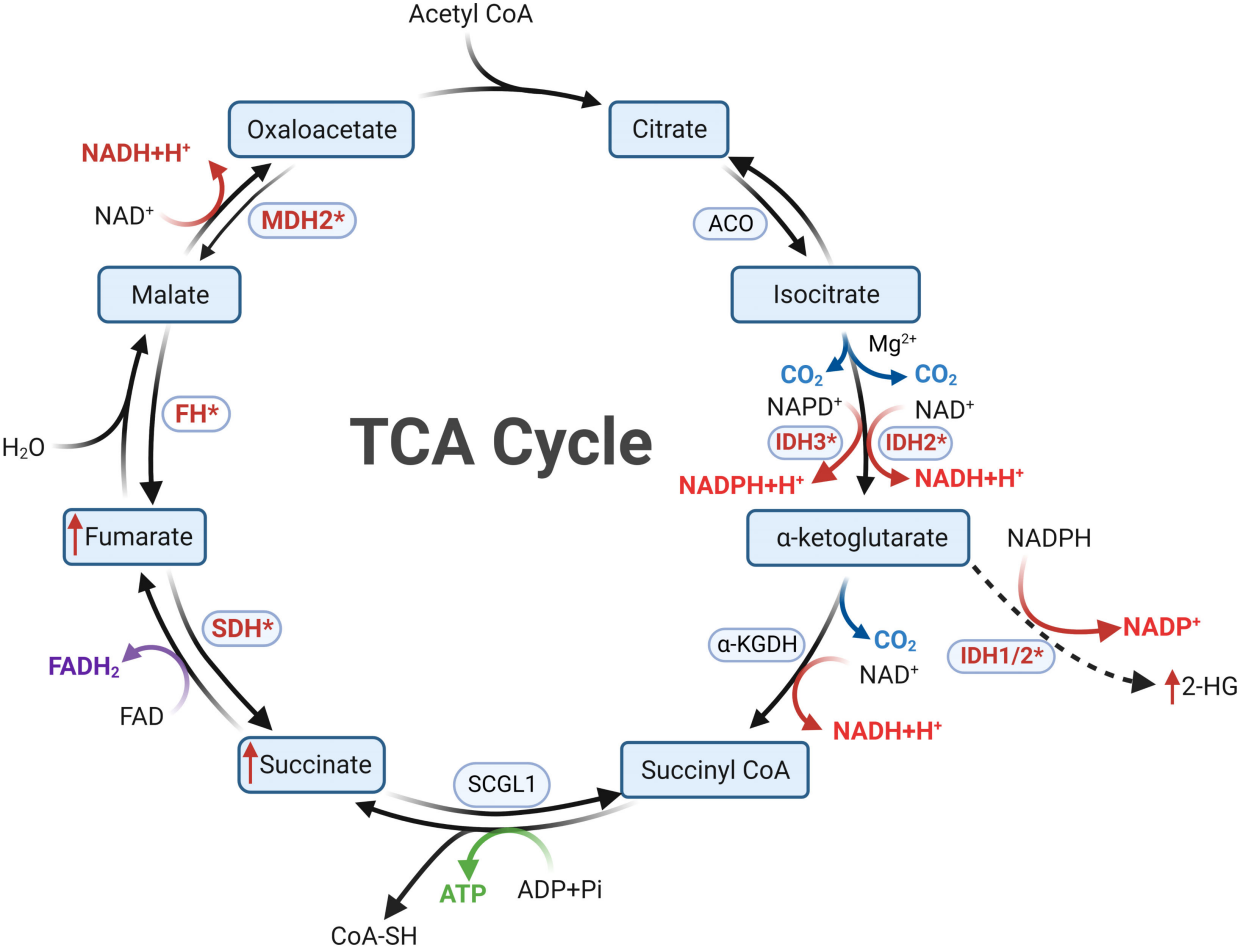
Metabolic reprogramming following mutations in tricarboxylic acid cycle enzymes is a key feature of Cluster 1a. In cells with normal mitochondrial function, the tricarboxylic acid cycle proceeds in a clockwise order. Mutations in SDHx and FH result in diminished substrate metabolism, leading to the accumulation of succinate and fumarate, respectively. Mutations in IDH confer the ability to convert αKG into 2-HG, resulting in 2-HG accumulation. Succinate, fumarate, and 2-HG all possess the capability to inhibit PHDs, thereby inducing a pseudohypoxic state. The oncogenic mechanisms following MDH mutations in PPGLs remain currently unclear.

A century ago, Otto Warburg’s seminal investigations unveiled a noteworthy phenomenon: even under aerobic conditions, tumor cells tend to favor glycolytic pathways for energy procurement over mitochondrial respiration, positing mitochondrial dysfunction as a plausible cause, known as the Warburg effect (52). Subsequent research has illuminated that mitochondrial dysfunction isn’t an obligatory trigger for tumorigenesis. Certain tumor cells adeptly generate energy through oxidative phosphorylation (OXPHOS) despite the mitochondrial milieu (53). Within this energetic landscape, the TCA cycle emerges as a pivotal conduit of mitochondrial energy metabolism. It orchestrates the oxidation of acetyl-CoA, transported to the mitochondria, into carbon dioxide, concomitantly releasing energy and reducing agents (NADH and FADH2) to facilitate subsequent OXPHOS (see **Figure 2**). Beyond its energy-contributing role, the TCA cycle acts as a nexus for intracellular carbohydrate, lipid, and amino acid metabolism, endowing other metabolic pathways with acetyl-CoA or diverse intermediary substrates (54, 55). Collectively, the nexus of tumorigenesis and the TCA cycle is unequivocally evident. Further probing into the intricate relationship between the TCA cycle and tumorigenesis holds the potential to unravel unique facets of tumor metabolism and unveil novel therapeutic targets.

Within the context of certain PPGLs, genetic perturbations affecting enzymes integral to the TCA cycle, such as SDH and FH, emerge as salient features (56). Since the initial discovery in 2000 of the correlation between SDHD germline mutations and familial paragangliomatosis, a growing compendium has cataloged mutations within at least twelve TCA cycle-related genes in the landscape of PPGLs (SDHB, SDH, SDHC, SDHD, SDHAF2, FH, IDH1, MDH2, SUCLG2, DLST, SLC25A11, and GOT2) (8, 57, 58). Among these, SDHx, FH, IDH1, and MDH2 have garnered relatively more attention, with mutations within SDHx and FH, in particular, emerging as pivotal drivers in the genesis and progression of PPGLs. Subsequent discourse delves into the myriad mutations within these four enzyme categories, expounding on their repercussions on intermediary metabolism.

## SDHx and succinate

Within the context of PPGLs, SDHx-related genetic mutations stand out as the most prevalent, accounting for approximately 15% (59). SDHx comprises four core subunits (SDHA, SDHB, SDHC, SDHD) and an assembly factor (SDHAF2) (8, 60). Among the five constituent molecules of SDHx, mutations associated with SDHD and SDHB are more commonly observed, while those affecting SDHA, SDHC, and SDHAF2 are relatively less frequent. In patients with SDHB mutations, approximately 50% experience metastatic progression, whereas patients with SDHD mutations typically manifest with multiple neck paragangliomas (8, 61–63).

SDHx plays a dual role by participating in both the TCA cycle, where it oxidizes succinate to fumarate, and serving as a constituent of mitochondrial complex II in the electron transport chain (ETC), catalyzing electron transfer to the ubiquinone pool (59). In the realm of PPGLs, SDHx serves as suppressors of tumorigenesis. When germline or somatic mutations occur, the functional integrity of succinate dehydrogenase is compromised, leading to interruptions in the TCA cycle and impairment of the electron transport chain. Following such disruption, the accumulation of succinate, a metabolic substrate of SDHx, occurs intracellularly. This accumulation is responsible for reduced metabolic product generation, leading to diminished mitochondrial energy production. Concurrently, tumor cells undergo metabolic reprogramming to satisfy the demands for crucial synthesis processes. In cells with SDHx functional defects, heightened glycolysis and enhanced citric acid cycle flux are observed, primarily to sustain aspartate requirements, crucial for protein and nucleic acid synthesis (64, 65). Additionally, the terminal product of compensatory upregulated glycolysis, lactate, has been demonstrated to stimulate tumor cell growth (66).

The accrual of succinate in cells with SDHx functional defects is deemed a primary mediator of SDH-associated tumorigenesis (67). Pathological succinate accumulation leads to its leakage from the mitochondrial matrix to the cytoplasm. As a competitor of α-ketoglutarate (αKG), succinate broadly inhibits αKG-dependent dioxygenases, including the Ten-Eleven Translocation (TET) DNA hydroxylases and Jumonji (JMJ) histone demethylases (KDMs). Consequently, a global hypermethylation characterized by the CpG island methylator phenotype (CIMP) emerges in tumor cells, inducing alterations in gene expression and facilitating tumorigenesis (60, 68). Furthermore, SDHx deficiency-induced succinate accumulation competitively inhibits the αKG -dependent PHDs family (PHD1-3) in the cytoplasm. This leads to the stabilization of HIFα under normoxic conditions, thereby contributing to the activation of the pseudohypoxia pathway (25, 69, 70). Notably, succinate serves not only as a substrate for mitochondrial SDHx but also as a product of cytoplasmic PHDs (33, 69, 71–74). Consequently, the accumulation of succinate, through negative feedback regulation, inhibits the activity of PHDs, resulting in further stabilization and activation of HIF complexes under normoxic conditions. Research by Celada et al. suggests that in SDHx mutation-driven PPGLs, diminished expression of PD-L1 and lower infiltration of cytotoxic T lymphocytes (CTLs) contribute to a ‘cold’ immunophenotype significantly associated with SDHx mutations (25). This hints at the potential of SDHx mutations to foster an immune-suppressive tumor microenvironment.

Furthermore, extracellular succinate secretion by tumor cells with SDH defects yields functional consequences (75). Extracellular succinate, upon binding to succinate receptor-1 (SUCNR-1), activates several oncogenic signaling molecules, such as ERK1/2, Akt, and AMPK, thereby enhancing tumor cell invasion and metastasis (75–77). Notably, Wu et al. found that the accumulated succinate in SDH-deficient tumor cells not only upregulates HIF-1A expression via the activation of the intracellular PI3K/Akt signaling pathway but also contributes to tumor invasion by activating macrophage surface SUCNR-1, subsequently inducing M1 polarization and promoting a malignant tumor phenotype (75, 78). Beyond immune cell interactions, secreted succinate from tumor cells binds to endothelial cell SUCNR-1, activating downstream STAT3-ERK1/2 in a HIF-independent manner and subsequently upregulating VEGF, thereby fostering angiogenesis.

## FH and fumarate

FH utilizes fumarate as a metabolic substrate, catalyzing its reversible hydration into malate (79). Mutations in FH result in reduced or loss of enzymatic activity, leading to the accumulation of high levels of fumarate (at millimolar levels). This accumulation profoundly alters mitochondrial function and cellular metabolism. Upon mitochondrial dysfunction due to FH mutations, cells undergo metabolic reprogramming, transitioning from mitochondrial oxidative respiration to cytoplasmic glycolysis. This metabolic shift also leads to a further reduction in carbon sources entering the mitochondria, which poses a risk of decreased membrane potential, potentially resulting in increased ROS and cell death. In this scenario, glutamine becomes an alternative carbon source to sustain the compromised TCA cycle, generating NADH for subsequent OXPHOS and ATP production to maintain mitochondrial membrane potential (80). To prevent the accumulation of other metabolic intermediates within this compensatory pathway, a portion of the glutamine-derived product is utilized for heme biosynthesis and degradation pathways, crucial for maintaining mitochondrial quality control and cell viability in FH-deficient cells (80, 81).

In addition to the metabolic reprogramming caused by FH deficiency and fumarate accumulation, these changes can also activate pro-oncogenic signaling pathways in FH-deficient cells. Fumarate can stabilize the majority of proteins through succination, a modification in which fumarate reacts with exposed cysteine residues on protein surfaces to form stable thiols. This leads to functional inactivation of proteins within the dynamic intracellular environment of tumor cells (82, 83). In a cellular environment characterized by elevated intracellular fumarate levels, succination of various oncoproteins promotes cell survival and proliferation. For example, fumarate succination of KEAP1 inactivates its ubiquitin ligase function, resulting in the stabilization and activation of its downstream target NRF2. As a transcription factor, NRF2 further upregulates the transcription of heme oxygenase 1 (HMOX1), a process crucial for maintaining heme metabolism. Additionally, iron-responsive element-binding protein 2 (IRP2) is a critical regulator of cellular iron metabolism, maintaining intracellular iron levels by inhibiting ferritin translation. Succination of IRP2 reduces its translational regulatory capacity on ferritin, leading to increased ferritin levels and decreased iron ions, disrupting intracellular iron homeostasis (84, 85). Furthermore, ferritin upregulates the expression of forkhead box protein M1 (FOXM1), promoting tumor cells to undergo mitosis (85).

Similar to succinate, fumarate also competitively inhibits PHDs, leading to increased stability of HIFs and inducing a pseudohypoxic state in tumorigenesis (86, 87). Thus, even in the context of mitochondrial defects in the energy metabolism chain, tumor cells maintain their malignant potential. Worth noting is that in FH-deficient tumor cells, activation of downstream pathways by HIFs results in significant upregulation of GLUT1, facilitating glucose uptake (86). Conversely, the expressions of pyruvate dehydrogenase kinase and lactate dehydrogenase are suppressed (88, 89). These changes in HIF-mediated metabolic genes lead to inhibition of mitochondrial oxidative metabolism, redirecting cells toward glycolysis and lactate synthesis, which hold essential significance in maintaining mitochondrial homeostasis.

## IDH and 2-HG

There are two forms of IDH in cells: IDH1/2 and IDH3, with IDH1/2 being NADP-dependent and IDH3 being NAD-dependent. While IDH1 and IDH2 share similarities in structure and function, they differ in subcellular localization. IDH1 is located in the cytoplasm and peroxisomes, whereas IDH2 is localized in the mitochondria. The function of IDH1/2 is to catalyze the oxidative decarboxylation of isocitrate to form αKG, simultaneously reducing NAD(P) to NAD(P)H (90). Unlike IDH1/2, IDH3 is found in the mitochondria and functions in the respiratory chain by catalyzing the forward decarboxylation of isocitrate to produce αKG (91). In 2010, Gaal and colleagues first identified a solitary IDH1 mutation in a case of sporadic carotid paraganglioma among 365 PPGLs specimens (92). Unfortunately, in another study by Yao and colleagues involving 104 PPGLs samples, no IDH1 mutations were detected (93). This suggests that pathogenic IDH mutations in PPGL are rare. Currently, there is a lack of authoritative research on the mechanisms by which IDH mutations contribute to PPGLs pathogenesis and progression, although the oncogenic mechanisms of IDH mutations have been extensively studied in diseases like gliomas and leukemias.

IDH1/2 mutations are considered gain-of-function oncogenic mutations. A single amino acid residue mutation in the IDH catalytic subunit prevents the conversion of isocitrate to α-ketoglutarate, while gaining new enzymatic activity to generate D-2HG from αKG (94–96). This leads to a significant accumulation of this oncogenic metabolite in cells. Simultaneously, D-2HG disrupts the balance of αKG-assisted dioxygenases, including histone lysine demethylases, TET DNA hydroxylases, and PHDs. For example, inhibition of histone lysine demethylase activity results in increased methylation levels of histone lysine residues in chromatin (91, 97, 98). Furthermore, D-2HG inhibits PHDs, thereby preventing the ubiquitination and degradation of HIFs, leading to their stabilization and accumulation within cells (99, 100). Consequently, this elevation in HIFs target gene transcription promotes the formation of a pro-tumorigenic immune microenvironment. Under physiological metabolic conditions, NADPH is a reduction product of IDH. After IDH mutation, reduced NADPH generation impairs the maintenance of the reduced glutathione (GSH) pool, causing a decrease in the ratio of GSH to GSSG and disrupting intracellular antioxidant system balance, resulting in increased ROS (98). However, in the context of elevated intracellular ROS due to IDH1 loss-of-function, HIF2α stabilization can be induced in a ROS-dependent manner (101).

On the other hand, IDH mutation alters its original catalytic pathway, resulting in reduced metabolic flux of αKG and NADPH in the cell, profoundly affecting cellular metabolic status (91). Interestingly, in contrast to the outcome of HIF signaling pathway activation observed after IDH mutation, studies suggest that tumor cells may release D-2HG into the tumor microenvironment, triggering instability of HIF-1α in immune cells (regulatory T cells and Th17 cells) of the immune microenvironment (102). This subsequently modulates the energy metabolism and anti-tumor immune function of immune cells, although the precise mechanisms remain unclear. In a study focusing on enantiomer-specific mechanisms, it was found that IDH1/2 mutant variants can convert 2-oxoglutarate (2-OG) to (R)-2HG rather than (S)-2HG. (R)-2HG can activate PHD activity, thereby reducing the stability and levels of HIFs (103). Overall, this complex interplay contributes to malignant transformation of normal cells and enables tumor cells to survive energy reprogramming (97, 98, 104, 105).

## MDH2

Malate dehydrogenase 2 (encoded by the MDH2 gene) is downstream of fumarase and functions to oxidize malate into oxaloacetate. This enzyme is also involved in the malate-aspartate shuttle, which is essential for cellular respiration. In recent years, MDH2 has been considered a potential susceptibility gene for PPGLs (106). Reported cases of PPGLs associated with MDH2 mutations are extremely rare, with only a few cases retrievable in databases since the initial report by Alberto et al. in 2015 (106–108). MDH2 variants have been identified in metastatic cases, with approximately 50% of cases estimated to progress to metastasis (106), suggesting a potential association between MDH2 polymorphic variations and the metastatic nature of PPGLs. However, the mechanisms underlying the relationship between MDH2 mutations and the onset and progression of PPGLs remain unclear. In a larger-scale study, germline mutations in MDH2 were found to account for about 0.6% of overall PPGLs, and the p.K314del variant’s potential pathogenicity in PPGLs was identified, possibly due to its impact on amino acid stability, although the specific mechanism is not yet understood (106).

Research has indicated that MDH2 mutations can promote HIFs stability by inhibiting PHDs, thereby facilitating pseudohypoxic responses in PPGLs (109–111). Conversely, some pharmacological studies have shown that LW6, an aroxyacetaminobenzoic acid analogue, can inhibit MDH1 activity. This leads to mitochondrial respiratory impairment, reducing cellular oxygen consumption and ATP production, resulting in elevated intracellular oxygen levels and triggering oxygen-dependent degradation of HIFs (112, 113).

## Conclusion and future perspectives

In summary, the intricate and complex interplay between metabolic enzyme mutations within the TCA cycle and the pseudohypoxic signaling has illuminated the potential mechanisms underlying the pathogenesis of PPGLs. The identification of key genetic mutations and their impact on critical metabolic intermediates such as succinate, fumarate, and 2-hydroxyglutarate underscores the significance of pseudo-hypoxia in promoting tumor initiation and progression. Under normoxic conditions, the activation of HIFs and their downstream effector pathways highlights the adaptive advantage of tumor cells within adverse microenvironments. Moreover, these mutations’ influence on immune cell communication patterns and the establishment of an immune-suppressive tumor microenvironment further underscores the intricacy of PPGLs development. Insights gained from understanding alterations in PPGLs metabolism serve as the foundation for developing precise and effective therapeutic strategies. Additionally, the broader implications of these findings extend to the realm of cellular metabolism and its pivotal role in tumorigenesis.

Looking ahead, delving deeper into the intricate network of metabolic reprogramming and pseudo-hypoxic signaling in PPGLs holds great promise. Elucidating the detailed molecular mechanisms by which metabolic enzyme mutations drive HIFs activation and pseudohypoxia is paramount for designing targeted and efficacious therapeutic interventions. Furthermore, investigating the crosstalk between pseudo-hypoxic signaling and other molecular clusters such as the WNT and kinase pathways may reveal further complexities and potential therapeutic targets. The advancements in techniques such as high-throughput sequencing and metabolomics analysis offer exciting opportunities to uncover the genetic landscape and metabolic adaptations driving PPGLs development. Integrating these comprehensive datasets with functional studies will contribute to a profound understanding of the disease and aid in the discovery of novel biomarkers for early diagnosis and prognosis.

To sum up, delving into the role of TCA cycle metabolic enzyme mutations and pseudohypoxic signaling in PPGLs not only enhances our comprehension of these rare neuroendocrine tumors but also provides broader insights into cancer biology and the field of metabolism. These insights ignite the potential for innovative therapeutic approaches and diagnostic methods, with the promise of significant clinical impact in the management of PPGLs and beyond.

## Author contributions

YXW: Writing – original draft, Writing – review & editing. BL: Conceptualization, Validation, Writing – review & editing. FL: Conceptualization, Validation, Writing – review & editing. YZ: Writing – review & editing. XG: Writing – review & editing. YSW: Funding acquisition, Supervision, Writing – review & editing. HZ: Funding acquisition, Supervision, Writing – review & editing.

## References

[1] Al Subhi AR Boyle V Elston MS. Systematic review: incidence of pheochromocytoma and paraganglioma over 70 years. J Endocrine Society (2022) 6(9):bvac105. doi: 10.1210/jendso/bvac105 PMC9334688 35919261

[2] Martinelli S Amore F Canu L Maggi M Rapizzi E. Tumour microenvironment in pheochromocytoma and paraganglioma. Front Endocrinol (2023) 14:1137456. doi: 10.3389/fendo.2023.1137456 PMC10073672 37033265

[3] Yamazaki Y Gao X Pecori A Nakamura Y Tezuka Y Omata K. Recent advances in histopathological and molecular diagnosis in pheochromocytoma and paraganglioma: challenges for predicting metastasis in individual patients. Front Endocrinol (2020) 11:587769. doi: 10.3389/fendo.2020.587769 PMC7652733 33193100

[4] Ebbehoj A Stochholm K Jacobsen SF Trolle C Jepsen P Robaczyk MG. Incidence and clinical presentation of pheochromocytoma and sympathetic paraganglioma: a population-based study. J Clin Endocrinol Metab (2021) 106(5):e2251–e61. doi: 10.1210/clinem/dgaa965 33479747

[5] Jimenez C Xu G Varghese J Graham PH Campbell MT Lu Y. New directions in treatment of metastatic or advanced pheochromocytomas and sympathetic paragangliomas: an american, contemporary, pragmatic approach. Curr Oncol Rep (2022) 24(1):89–98. doi: 10.1007/s11912-022-01197-0 35061191

[6] Mete O Asa SL Gill AJ Kimura N de Krijger RR Tischler A. Overview of the 2022 WHO classification of paragangliomas and pheochromocytomas. Endocrine Pathol (2022) 33(1):90–114. doi: 10.1007/s12022-022-09704-6 35285002

[7] Tanabe A Naruse M. Recent advances in the management of pheochromocytoma and paraganglioma. Hypertension Res Off J Japanese Soc Hypertension (2020) 43(11):1141–51. doi: 10.1038/s41440-020-0531-0 32778780

[8] Buffet A Burnichon N Favier J Gimenez-Roqueplo AP. An overview of 20 years of genetic studies in pheochromocytoma and paraganglioma. Best Pract Res Clin Endocrinol Metab (2020) 34(2):101416. doi: 10.1016/j.beem.2020.101416 32295730

[9] Wang K Crona J Beuschlein F Grossman AB Pacak K Nölting S. Targeted therapies in pheochromocytoma and paraganglioma. J Clin Endocrinol Metab (2022) 107(11):2963–72. doi: 10.1210/clinem/dgac471 PMC9923802 35973976

[10] Kantorovich V King KS Pacak K. SDH-related pheochromocytoma and paraganglioma. Best Pract Res Clin Endocrinol Metab (2010) 24(3):415–24. doi: 10.1016/j.beem.2010.04.001 PMC2939070 20833333

[11] Jochmanová I Yang C Zhuang Z Pacak K. Hypoxia-inducible factor signaling in pheochromocytoma: turning the rudder in the right direction. J Natl Cancer Institute (2013) 105(17):1270–83. doi: 10.1093/jnci/djt201 PMC3888279 23940289

[12] Alrezk R Suarez A Tena I Pacak K. Update of pheochromocytoma syndromes: genetics, biochemical evaluation, and imaging. Front Endocrinol (2018) 9:515. doi: 10.3389/fendo.2018.00515 PMC6277481 30538672

[13] Eid M Foukal J Sochorová D Tuček Š Starý K Kala Z. Management of pheochromocytomas and paragangliomas: Review of current diagnosis and treatment options. Cancer Med (2023) 12(13):13942–57. doi: 10.1002/cam4.6010 PMC10358258 37145019

[14] Nölting S Bechmann N Taieb D Beuschlein F Fassnacht M Kroiss M. Personalized management of pheochromocytoma and paraganglioma. Endocrine Rev (2022) 43(2):199–239. doi: 10.1210/endrev/bnab019 34147030 PMC8905338

[15] Lenders JWM Kerstens MN Amar L Prejbisz A Robledo M Taieb D. Genetics, diagnosis, management and future directions of research of phaeochromocytoma and paraganglioma: a position statement and consensus of the Working Group on Endocrine Hypertension of the European Society of Hypertension. J Hypertension (2020) 38(8):1443–56. doi: 10.1097/HJH.0000000000002438 PMC7486815 32412940

[16] Mazzaglia PJ. Hereditary pheochromocytoma and paraganglioma. J Surg Oncol (2012) 106(5):580–5. doi: 10.1002/jso.23157 22648936

[17] Lenders JW Duh QY Eisenhofer G Gimenez-Roqueplo AP Grebe SK Murad MH. Pheochromocytoma and paraganglioma: an endocrine society clinical practice guideline. J Clin Endocrinol Metab (2014) 99(6):1915–42. doi: 10.1210/jc.2014-1498 24893135

[18] Favier J Amar L Gimenez-Roqueplo AP. Paraganglioma and phaeochromocytoma: from genetics to personalized medicine. Nat Rev Endocrinol (2015) 11(2):101–11. doi: 10.1038/nrendo.2014.188 25385035

[19] Muth A Crona J Gimm O Elmgren A Filipsson K Stenmark Askmalm M. Genetic testing and surveillance guidelines in hereditary pheochromocytoma and paraganglioma. J Internal Med (2019) 285(2):187–204. doi: 10.1111/joim.12869 30536464

[20] Erlic Z Rybicki L Peczkowska M Golcher H Kann PH Brauckhoff M. Clinical predictors and algorithm for the genetic diagnosis of pheochromocytoma patients. Clin Cancer Res (2009) 15(20):6378–85. doi: 10.1158/1078-0432.CCR-09-1237 19825962

[21] Jha A de Luna K Balili CA Millo C Paraiso CA Ling A. Clinical, diagnostic, and treatment characteristics of SDHA-related metastatic pheochromocytoma and paraganglioma. Front Oncol (2019) 9:53. doi: 10.3389/fonc.2019.00053 30854332 PMC6395427

[22] Else T Marvin ML Everett JN Gruber SB Arts HA Stoffel EM. The clinical phenotype of SDHC-associated hereditary paraganglioma syndrome (PGL3). J Clin Endocrinol Metab (2014) 99(8):E1482–6. doi: 10.1210/jc.2013-3853 PMC4121019 24758179

[23] Hescot S Curras-Freixes M Deutschbein T van Berkel A Vezzosi D Amar L. Prognosis of Malignant pheochromocytoma and paraganglioma (MAPP-prono study): a european network for the study of adrenal tumors retrospective study. J Clin Endocrinol Metab (2019) 104(6):2367–74. doi: 10.1210/jc.2018-01968 30715419

[24] Taïeb D Kaliski A Boedeker CC Martucci V Fojo T Adler JR Jr.. Current approaches and recent developments in the management of head and neck paragangliomas. Endocrine Rev (2014) 35(5):795–819. doi: 10.1210/er.2014-1026 25033281 PMC4167435

[25] Celada L Cubiella T San-Juan-Guardado J Gutiérrez G Beiguela B Rodriguez R. Pseudohypoxia in paraganglioma and pheochromocytoma is associated with an immunosuppressive phenotype. J Pathol (2023) 259(1):103–14. doi: 10.1002/path.6026 PMC10107524 36314599

[26] Bratslavsky G Sudarshan S Neckers L Linehan WM. Pseudohypoxic pathways in renal cell carcinoma. Clin Cancer Res (2007) 13(16):4667–71. doi: 10.1158/1078-0432.CCR-06-2510 17699843

[27] Talty R Olino K. Metabolism of innate immune cells in cancer. Cancers (2021) 13(4). doi: 10.3390/cancers13040904 PMC7927092 33670082

[28] Mohlin S Wigerup C Jögi A Påhlman S. Hypoxia, pseudohypoxia and cellular differentiation. Exp Cell Res (2017) 356(2):192–6. doi: 10.1016/j.yexcr.2017.03.007 28284840

[29] Brereton CJ Yao L Davies ER Zhou Y Vukmirovic M Bell JA. Pseudohypoxic HIF pathway activation dysregulates collagen structure-function in human lung fibrosis. eLife (2022) 11. doi: 10.7554/eLife.69348 PMC8860444 35188460

[30] Menendez JA Alarcón T Joven J. Gerometabolites: the pseudohypoxic aging side of cancer oncometabolites. Cell Cycle (Georgetown Tex) (2014) 13(5):699–709. doi: 10.4161/cc.28079 24526120 PMC3979906

[31] Jochmanova I Pacak K. Genomic landscape of pheochromocytoma and paraganglioma. Trends Cancer (2018) 4(1):6–9. doi: 10.1016/j.trecan.2017.11.001 29413423 PMC5819363

[32] Redlich A Pamporaki C Lessel L Frühwald MC Vorwerk P Kuhlen M. Pseudohypoxic pheochromocytomas and paragangliomas dominate in children. Pediatr Blood Cancer (2021) 68(7):e28981. doi: 10.1002/pbc.28981 33682326

[33] Jhawar S Arakawa Y Kumar S Varghese D Kim YS Roper N. New insights on the genetics of pheochromocytoma and paraganglioma and its clinical implications. Cancers (2022) 14(3). doi: 10.3390/cancers14030594 PMC8833412 35158861

[34] Ilanchezhian M Jha A Pacak K Del Rivero J. Emerging treatments for advanced/metastatic pheochromocytoma and paraganglioma. Curr Treat Options Oncol (2020) 21(11):85. doi: 10.1007/s11864-020-00787-z 32862332 PMC7456409

[35] Pouysségur J Dayan F Mazure NM. Hypoxia signalling in cancer and approaches to enforce tumour regression. Nature (2006) 441(7092):437–43. doi: 10.1038/nature04871 16724055

[36] Lonser RR Glenn GM Walther M Chew EY Libutti SK Linehan WM. von hippel-lindau disease. Lancet (London England) (2003) 361(9374):2059–67. doi: 10.1016/S0140-6736(03)13643-4 12814730

[37] Kaelin WG Jr. Ratcliffe PJ. Oxygen sensing by metazoans: the central role of the HIF hydroxylase pathway. Mol Cell (2008) 30(4):393–402. doi: 10.1016/j.molcel.2008.04.009 18498744

[38] Shen C Kaelin WG Jr. The VHL/HIF axis in clear cell renal carcinoma. Semin Cancer Biol (2013) 23(1):18–25. doi: 10.1016/j.semcancer.2012.06.001 22705278 PMC3663044

[39] Jang M Kim SS Lee J. Cancer cell metabolism: implications for therapeutic targets. Exp Mol Med (2013) 45(10):e45. doi: 10.1038/emm.2013.85 24091747 PMC3809361

[40] Watts D Jaykar MT Bechmann N Wielockx B. Hypoxia signaling pathway: a central mediator in endocrine tumors. Front Endocrinol (2022) 13:1103075. doi: 10.3389/fendo.2022.1103075 PMC9868855 36699028

[41] Kaelin WG Jr. Von Hippel-Lindau disease: insights into oxygen sensing, protein degradation, and cancer. J Clin Invest (2022) 132(18). doi: 10.1172/JCI162480 PMC9479583 36106637

[42] Burnichon N Vescovo L Amar L Libé R de Reynies A Venisse A. Integrative genomic analysis reveals somatic mutations in pheochromocytoma and paraganglioma. Hum Mol Genet (2011) 20(20):3974–85. doi: 10.1093/hmg/ddr324 21784903

[43] Peng S Zhang J Tan X Huang Y Xu J Silk N. The VHL/HIF axis in the development and treatment of pheochromocytoma/paraganglioma. Front Endocrinol (2020) 11:586857. doi: 10.3389/fendo.2020.586857 PMC7732471 33329393

[44] Ong KR Woodward ER Killick P Lim C Macdonald F Maher ER. Genotype-phenotype correlations in von Hippel-Lindau disease. Hum Mutation (2007) 28(2):143–9. doi: 10.1002/humu.20385 17024664

[45] Liu SJ Wang JY Peng SH Li T Ning XH Hong BA. Genotype and phenotype correlation in von Hippel-Lindau disease based on alteration of the HIF-α binding site in VHL protein. Genet Med (2018) 20(10):1266–73. doi: 10.1038/gim.2017.261 29595810

[46] Andreasson A Kiss NB Caramuta S Sulaiman L Svahn F Bäckdahl M. The VHL gene is epigenetically inactivated in pheochromocytomas and abdominal paragangliomas. Epigenetics (2013) 8(12):1347–54. doi: 10.4161/epi.26686 PMC3933494 24149047

[47] Lorenzo FR Yang C Ng Tang Fui M Vankayalapati H Zhuang Z Huynh T. A novel EPAS1/HIF2A germline mutation in a congenital polycythemia with paraganglioma. J Mol Med (Berlin Germany) (2013) 91(4):507–12. doi: 10.1007/s00109-012-0967-z PMC3570726 23090011

[48] Ladroue C Carcenac R Leporrier M Gad S Le Hello C Galateau-Salle F. PHD2 mutation and congenital erythrocytosis with paraganglioma. New Engl J Med (2008) 359(25):2685–92. doi: 10.1056/NEJMoa0806277 19092153

[49] Richter S Peitzsch M Rapizzi E Lenders JW Qin N de Cubas AA. Krebs cycle metabolite profiling for identification and stratification of pheochromocytomas/paragangliomas due to succinate dehydrogenase deficiency. J Clin Endocrinol Metab (2014) 99(10):3903–11. doi: 10.1210/jc.2014-2151 PMC4184070 25014000

[50] Ma X Li M Tong A Wang F Cui Y Zhang X. Genetic and clinical profiles of pheochromocytoma and paraganglioma: A single center study. Front Endocrinol (2020) 11:574662. doi: 10.3389/fendo.2020.574662 PMC7761866 33362715

[51] Richter S Garrett TJ Bechmann N Clifton-Bligh RJ Ghayee HK. Metabolomics in paraganglioma: applications and perspectives from genetics to therapy. Endocrine-related Cancer (2023) 30(6). doi: 10.1530/ERC-22-0376 PMC10228374 36897220

[52] Cascón A Remacha L Calsina B Robledo M. Pheochromocytomas and paragangliomas: bypassing cellular respiration. Cancers (2019) 11(5). doi: 10.3390/cancers11050683 PMC6562521 31100940

[53] Eniafe J Jiang S. The functional roles of TCA cycle metabolites in cancer. Oncogene (2021) 40(19):3351–63. doi: 10.1038/s41388-020-01639-8 33864000

[54] Owen OE Kalhan SC Hanson RW. The key role of anaplerosis and cataplerosis for citric acid cycle function. J Biol Chem (2002) 277(34):30409–12. doi: 10.1074/jbc.R200006200 12087111

[55] Haddad A Mohiuddin SS. Biochemistry, Citric Acid Cycle. StatPearls. Treasure Island (FL) ineligible companies. Disclosure: Shamim Mohiuddin declares no relevant financial relationships with ineligible companies. StatPearls Publishing Copyright © 2023, StatPearls Publishing LLC (2023).

[56] Mannelli M Rapizzi E Fucci R Canu L Ercolino T Luconi M. 15 YEARS OF PARAGANGLIOMA: metabolism and pheochromocytoma/paraganglioma. Endocrine-related Cancer (2015) 22(4):T83–90. doi: 10.1530/ERC-15-0215 26113605

[57] Cascón A Calsina B Monteagudo M Mellid S Díaz-Talavera A Currás-Freixes M. Genetic bases of pheochromocytoma and paraganglioma. J Mol Endocrinol (2023) 70(3). doi: 10.1530/JME-22-0167 36520714

[58] Hadrava Vanova K Pang Y Krobova L Kraus M Nahacka Z Boukalova S. Germline SUCLG2 variants in patients with pheochromocytoma and paraganglioma. J Natl Cancer Institute (2022) 114(1):130–8. doi: 10.1093/jnci/djab158 PMC8755484 34415331

[59] Gill AJ. Succinate dehydrogenase (SDH)-deficient neoplasia. Histopathology (2018) 72(1):106–16. doi: 10.1111/his.13277 29239034

[60] Moog S Lussey-Lepoutre C Favier J. Epigenetic and metabolic reprogramming of SDH-deficient paragangliomas. Endocrine-related Cancer (2020) 27(12):R451–r63. doi: 10.1530/ERC-20-0346 33112834

[61] Benn DE Richardson AL Marsh DJ Robinson BG. Genetic testing in pheochromocytoma- and paraganglioma-associated syndromes. Ann New York Acad Sci (2006) 1073:104–11. doi: 10.1196/annals.1353.011 17102077

[62] Amar L Baudin E Burnichon N Peyrard S Silvera S Bertherat J. Succinate dehydrogenase B gene mutations predict survival in patients with Malignant pheochromocytomas or paragangliomas. J Clin Endocrinol Metab (2007) 92(10):3822–8. doi: 10.1210/jc.2007-0709 17652212

[63] Taïeb D Wanna GB Ahmad M Lussey-Lepoutre C Perrier ND Nölting S. Clinical consensus guideline on the management of phaeochromocytoma and paraganglioma in patients harbouring germline SDHD pathogenic variants. Lancet Diabetes Endocrinol (2023) 11(5):345–61. doi: 10.1016/S2213-8587(23)00038-4 PMC10182476 37011647

[64] Cardaci S Zheng L MacKay G van den Broek NJ MacKenzie ED Nixon C. Pyruvate carboxylation enables growth of SDH-deficient cells by supporting aspartate biosynthesis. Nat Cell Biol (2015) 17(10):1317–26. doi: 10.1038/ncb3233 PMC4591470 26302408

[65] Lussey-Lepoutre C Hollinshead KE Ludwig C Menara M Morin A Castro-Vega LJ. Loss of succinate dehydrogenase activity results in dependency on pyruvate carboxylation for cellular anabolism. Nat Commun (2015) 6:8784. doi: 10.1038/ncomms9784 26522426 PMC4632646

[66] Faubert B Li KY Cai L Hensley CT Kim J Zacharias LG. Lactate metabolism in human lung tumors. Cell (2017) 171(2):358–71.e9. doi: 10.1016/j.cell.2017.09.019 28985563 PMC5684706

[67] Pollard PJ Brière JJ Alam NA Barwell J Barclay E Wortham NC. Accumulation of Krebs cycle intermediates and over-expression of HIF1alpha in tumours which result from germline FH and SDH mutations. Hum Mol Genet (2005) 14(15):2231–9. doi: 10.1093/hmg/ddi227 15987702

[68] Liu Y Pang Y Zhu B Uher O Caisova V Huynh TT. Therapeutic targeting of SDHB-mutated pheochromocytoma/paraganglioma with pharmacologic ascorbic acid. Clin Cancer Res (2020) 26(14):3868–80. doi: 10.1158/1078-0432.CCR-19-2335 PMC7443979 32152203

[69] King A Selak MA Gottlieb E. Succinate dehydrogenase and fumarate hydratase: linking mitochondrial dysfunction and cancer. Oncogene (2006) 25(34):4675–82. doi: 10.1038/sj.onc.1209594 16892081

[70] Kuo CC Wu JY Wu KK. Cancer-derived extracellular succinate: a driver of cancer metastasis. J Biomed Science (2022) 29(1):93. doi: 10.1186/s12929-022-00878-z PMC9641777 36344992

[71] Selak MA Armour SM MacKenzie ED Boulahbel H Watson DG Mansfield KD. Succinate links TCA cycle dysfunction to oncogenesis by inhibiting HIF-alpha prolyl hydroxylase. Cancer Cell (2005) 7(1):77–85. doi: 10.1016/j.ccr.2004.11.022 15652751

[72] Singleton DC Macann A Wilson WR. Therapeutic targeting of the hypoxic tumour microenvironment. Nat Rev Clin Oncol (2021) 18(12):751–72. doi: 10.1038/s41571-021-00539-4 34326502

[73] Chen X Sunkel B Wang M Kang S Wang T Gnanaprakasam JNR. Succinate dehydrogenase/complex II is critical for metabolic and epigenetic regulation of T cell proliferation and inflammation. Sci Immunol (2022) 7(70):eabm8161. doi: 10.1126/sciimmunol.abm8161 35486677 PMC9332111

[74] Germanova E Khmil N Pavlik L Mikheeva I Mironova G Lukyanova L. The role of mitochondrial enzymes, succinate-coupled signaling pathways and mitochondrial ultrastructure in the formation of urgent adaptation to acute hypoxia in the myocardium. Int J Mol Sci (2022) 23(22). doi: 10.3390/ijms232214248 PMC9696391 36430733

[75] Wu JY Huang TW Hsieh YT Wang YF Yen CC Lee GL. Cancer-derived succinate promotes macrophage polarization and cancer metastasis *via* succinate receptor. Mol Cell (2020) 77(2):213–27.e5. doi: 10.1016/j.molcel.2019.10.023 31735641

[76] Matlac DM Hadrava Vanova K Bechmann N Richter S Folberth J Ghayee HK. Succinate mediates tumorigenic effects *via* succinate receptor 1: potential for new targeted treatment strategies in succinate dehydrogenase deficient paragangliomas. Front Endocrinol (2021) 12:589451. doi: 10.3389/fendo.2021.589451 PMC7994772 33776908

[77] de Castro Fonseca M Aguiar CJ da Rocha Franco JA Gingold RN Leite MF. GPR91: expanding the frontiers of Krebs cycle intermediates. Cell Communication Signaling CCS (2016) 14:3. doi: 10.1186/s12964-016-0126-1 26759054 PMC4709936

[78] Trauelsen M Hiron TK Lin D Petersen JE Breton B Husted AS. Extracellular succinate hyperpolarizes M2 macrophages through SUCNR1/GPR91-mediated Gq signaling. Cell Rep (2021) 35(11):109246. doi: 10.1016/j.celrep.2021.109246 34133934

[79] Frezza C. Mitochondrial metabolites: undercover signalling molecules. Interface Focus (2017) 7(2):20160100. doi: 10.1098/rsfs.2016.0100 28382199 PMC5311903

[80] Frezza C Zheng L Folger O Rajagopalan KN MacKenzie ED Jerby L. Haem oxygenase is synthetically lethal with the tumour suppressor fumarate hydratase. Nature (2011) 477(7363):225–8. doi: 10.1038/nature10363 21849978

[81] Zorova LD Popkov VA Plotnikov EY Silachev DN Pevzner IB Jankauskas SS. Mitochondrial membrane potential. Analytical Biochem (2018) 552:50–9. doi: 10.1016/j.ab.2017.07.009 PMC5792320 28711444

[82] Bardella C El-Bahrawy M Frizzell N Adam J Ternette N Hatipoglu E. Aberrant succination of proteins in fumarate hydratase-deficient mice and HLRCC patients is a robust biomarker of mutation status. J Pathol (2011) 225(1):4–11. doi: 10.1002/path.2932 21630274

[83] Alderson NL Wang Y Blatnik M Frizzell N Walla MD Lyons TJ. S-(2-Succinyl)cysteine: a novel chemical modification of tissue proteins by a Krebs cycle intermediate. Arch Biochem Biophysics (2006) 450(1):1–8. doi: 10.1016/j.abb.2006.03.005 16624247

[84] Anderson CP Shen M Eisenstein RS Leibold EA. Mammalian iron metabolism and its control by iron regulatory proteins. Biochim Biophys Acta (2012) 1823(9):1468–83. doi: 10.1016/j.bbamcr.2012.05.010 PMC3675657 22610083

[85] Kerins MJ Vashisht AA Liang BX Duckworth SJ Praslicka BJ Wohlschlegel JA. Fumarate mediates a chronic proliferative signal in fumarate hydratase-inactivated cancer cells by increasing transcription and translation of ferritin genes. Mol Cell Biol (2017) 37(11). doi: 10.1128/MCB.00079-17 PMC5440649 28289076

[86] Isaacs JS Jung YJ Mole DR Lee S Torres-Cabala C Chung YL. HIF overexpression correlates with biallelic loss of fumarate hydratase in renal cancer: novel role of fumarate in regulation of HIF stability. Cancer Cell (2005) 8(2):143–53. doi: 10.1016/j.ccr.2005.06.017 16098467

[87] Frezza C Pollard PJ Gottlieb E. Inborn and acquired metabolic defects in cancer. J Mol Med (Berlin Germany) (2011) 89(3):213–20. doi: 10.1007/s00109-011-0728-4 PMC3043233 21301796

[88] Kim JW Tchernyshyov I Semenza GL Dang CV. HIF-1-mediated expression of pyruvate dehydrogenase kinase: a metabolic switch required for cellular adaptation to hypoxia. Cell Metab (2006) 3(3):177–85. doi: 10.1016/j.cmet.2006.02.002 16517405

[89] Xie H Valera VA Merino MJ Amato AM Signoretti S Linehan WM. LDH-A inhibition, a therapeutic strategy for treatment of hereditary leiomyomatosis and renal cell cancer. Mol Cancer Ther (2009) 8(3):626–35. doi: 10.1158/1535-7163.MCT-08-1049 PMC2671637 19276158

[90] Pillai S Gopalan V Smith RA Lam AK. Updates on the genetics and the clinical impacts on phaeochromocytoma and paraganglioma in the new era. Crit Rev Oncol/Hematol (2016) 100:190–208. doi: 10.1016/j.critrevonc.2016.01.022 26839173

[91] Waitkus MS Diplas BH Yan H. Isocitrate dehydrogenase mutations in gliomas. Neuro-Oncology (2016) 18(1):16–26. doi: 10.1093/neuonc/nov136 26188014 PMC4677412

[92] Gaal J Burnichon N Korpershoek E Roncelin I Bertherat J Plouin PF. Isocitrate dehydrogenase mutations are rare in pheochromocytomas and paragangliomas. J Clin Endocrinol Metab (2010) 95(3):1274–8. doi: 10.1210/jc.2009-2170 19915015

[93] Yao L Schiavi F Cascon A Qin Y Inglada-Pérez L King EE. Spectrum and prevalence of FP/TMEM127 gene mutations in pheochromocytomas and paragangliomas. Jama (2010) 304(23):2611–9. doi: 10.1001/jama.2010.1830 21156949

[94] Notarangelo G Spinelli JB Perez EM Baker GJ Kurmi K Elia I. Oncometabolite d-2HG alters T cell metabolism to impair CD8(+) T cell function. Sci (New York NY) (2022) 377(6614):1519–29. doi: 10.1126/science.abj5104 PMC9629749 36173860

[95] Ward PS Patel J Wise DR Abdel-Wahab O Bennett BD Coller HA. The common feature of leukemia-associated IDH1 and IDH2 mutations is a neomorphic enzyme activity converting α-ketoglutarate to 2-hydroxyglutarate. Cancer Cell (2010) 17(3):225–34. doi: 10.1016/j.ccr.2010.01.020 PMC2849316 20171147

[96] Dang L White DW Gross S Bennett BD Bittinger MA Driggers EM. Cancer-associated IDH1 mutations produce 2-hydroxyglutarate. Nature (2009) 462(7274):739–44. doi: 10.1038/nature08617 PMC2818760 19935646

[97] Zhao S Lin Y Xu W Jiang W Zha Z Wang P. Glioma-derived mutations in IDH1 dominantly inhibit IDH1 catalytic activity and induce HIF-1alpha. Sci (New York NY) (2009) 324(5924):261–5. doi: 10.1126/science.1170944 PMC3251015 19359588

[98] Laurenti G Tennant DA. Isocitrate dehydrogenase (IDH), succinate dehydrogenase (SDH), fumarate hydratase (FH): three players for one phenotype in cancer? Biochem Soc Trans (2016) 44(4):1111–6. doi: 10.1042/BST20160099 27528759

[99] Keith B Johnson RS Simon MC. HIF1α and HIF2α: sibling rivalry in hypoxic tumour growth and progression. Nat Rev Cancer (2011) 12(1):9–22. doi: 10.1042/BST20160099 22169972 PMC3401912

[100] Huang J Yu J Tu L Huang N Li H Luo Y. Isocitrate dehydrogenase mutations in glioma: from basic discovery to therapeutics development. Front Oncol (2019) 9:506. doi: 10.3389/fonc.2019.00506 31263678 PMC6584818

[101] Wang Y Agarwal E Bertolini I Ghosh JC Seo JH Altieri DC. IDH2 reprograms mitochondrial dynamics in cancer through a HIF-1α-regulated pseudohypoxic state. FASEB J (2019) 33(12):13398–411. doi: 10.1096/fj.201901366R PMC6894043 31530011

[102] Böttcher M Renner K Berger R Mentz K Thomas S Cardenas-Conejo ZE. D-2-hydroxyglutarate interferes with HIF-1α stability skewing T-cell metabolism towards oxidative phosphorylation and impairing Th17 polarization. Oncoimmunology (2018) 7(7):e1445454. doi: 10.1096/fj.201901366R 29900057 PMC5993507

[103] Koivunen P Lee S Duncan CG Lopez G Lu G Ramkissoon S. Transformation by the (R)-enantiomer of 2-hydroxyglutarate linked to EGLN activation. Nature (2012) 483(7390):484–8. doi: 10.1038/nature10898 PMC3656605 22343896

[104] Velasco-Hernandez T Hyrenius-Wittsten A Rehn M Bryder D Cammenga J. HIF-1α can act as a tumor suppressor gene in murine acute myeloid leukemia. Blood (2014) 124(24):3597–607. doi: 10.1182/blood-2014-04-567065 25267197

[105] Semukunzi H Roy D Li H Khan GJ Lyu X Yuan S. IDH mutations associated impact on related cancer epidemiology and subsequent effect toward HIF-1α. Biomed Pharmacother (2017) 89:805–11. doi: 10.1016/j.biopha.2017.02.083 28273642

[106] Calsina B Currás-Freixes M Buffet A Pons T Contreras L Letón R. Role of MDH2 pathogenic variant in pheochromocytoma and paraganglioma patients. Genet Med (2018) 20(12):1652–62. doi: 10.1038/s41436-018-0068-7 PMC7456538 30008476

[107] Cascón A Comino-Méndez I Currás-Freixes M de Cubas AA Contreras L Richter S. Whole-exome sequencing identifies MDH2 as a new familial paraganglioma gene. J Natl Cancer Institute (2015) 107(5). doi: 10.1093/jnci/djv053 25766404

[108] Mellid S Gil E Letón R Caleiras E Honrado E Richter S. Co-occurrence of mutations in NF1 and other susceptibility genes in pheochromocytoma and paraganglioma. Front Endocrinol (2022) 13:1070074. doi: 10.3389/fendo.2022.1070074 PMC9905101 36760809

[109] Jochmanová I Zhuang Z Pacak K. Pheochromocytoma: gasping for air. Hormones Cancer (2015) 6(5-6):191–205. doi: 10.1007/s12672-015-0231-4 26138106 PMC10355934

[110] Mercado-Asis LB Wolf KI Jochmanova I Taïeb D. Pheochromocytoma: a genetic and diagnostic update. Endocrine Pract (2018) 24(1):78–90. doi: 10.4158/EP-2017-0057 29144820

[111] Toledo RA Jimenez C Armaiz-Pena G Arenillas C Capdevila J Dahia PLM. Hypoxia-inducible factor 2 alpha (HIF2α) inhibitors: targeting genetically driven tumor hypoxia. Endocrine Rev (2023) 44(2):312–22. doi: 10.1210/endrev/bnac025 PMC10216878 36301191

[112] Naik R Won M Ban HS Bhattarai D Xu X Eo Y. Synthesis and structure-activity relationship study of chemical probes as hypoxia induced factor-1α/malate dehydrogenase 2 inhibitors. J Medicinal Chem (2014) 57(22):9522–38. doi: 10.1021/jm501241g 25356789

[113] Ban HS Xu X Jang K Kim I Kim BK Lee K. A novel malate dehydrogenase 2 inhibitor suppresses hypoxia-inducible factor-1 by regulating mitochondrial respiration. PloS One (2016) 11(9):e0162568. doi: 10.1371/journal.pone.0162568 27611801 PMC5017629

